# Composite healthy lifestyle, socioeconomic deprivation, and mental well-being during the COVID-19 pandemic: a prospective analysis

**DOI:** 10.1038/s41380-023-02338-y

**Published:** 2023-12-19

**Authors:** Gang Hu, Huibo Qin, Binbin Su, Yanping Bao, Zhengting Liang, Yunhe Wang

**Affiliations:** 1 School of Health Management (Health Management Center), Xinjiang Medical University, Urumqi, China; 2 Quality Control Department of Liaocheng People’s Hospital, Jinan, Shandong China; 3 School of Population Medicine and Public Health, Chinese Academy of Medical Sciences/Peking Union Medical College, Beijing, China; 4 National Institute on Drug Dependence, Peking University, Beijing, China; 5 School of Public Health, Peking University, Beijing, China; 6 School of Traditional Chinese Medicine, Xinjiang Medical University, Urumqi, China; 7 Nuffield Department of Population Health, University of Oxford, Oxford, UK

**Keywords:** Psychiatric disorders, Depression

## Abstract

The adverse psychological and social impacts of COVID-19 pandemic are well characterized, but the role of composite, modifiable lifestyle factors that may interact to mitigate these impacts is not. The effect of socioeconomic deprivation on these lifestyle risks also remains unclear. Based on a nationally representative, longitudinal cohort, we assessed the association between a combination of pre-pandemic lifestyle factors and mental health conditions during pandemic, and the contribution of deprivation to it. Composite lifestyle factors included BMI, smoking status, alcohol consumption, physical activity, sedentary time, sleep duration, and fruit and vegetable intake, with lifestyle scores and lifestyle categories calculated for each participant. Symptoms of depression and anxiety, and personal well-being were assessed by validated scales during the pandemic. Socioeconomic deprivation was characterized by both individual-level (income, wealth, and education) and group-level factors (Index of Multiple Deprivation). Of the 5049 eligible participants (mean [SD] age, 68.1 [10.9] years; 57.2% were female) included in the study, 41.6% followed a favorable lifestyle, 48.9% followed an intermediate lifestyle, and 9.5% followed an unfavorable lifestyle. Compared with favorable lifestyle category, participants in the intermediate and unfavorable lifestyle category were at increased risk of mental health conditions, with the hazard ratio (HR) for trend per increment change towards unfavorable category of 1.17 (95% CI 1.09–1.26) for depression, 1.23 (1.07–1.42) for anxiety, and 1.39 (1.20–1.61) for low well-being. A significant trend of lower risk for mental health conditions with increasing number of healthy lifestyle factors was observed (*P* < 0.001 for trend). There were no significant interactions between lifestyle factors and socioeconomic deprivation for any of the outcomes, with similar HRs for trend per one increment change in lifestyle category observed in each deprivation group. Compared with those in the least deprived group with favorable lifestyle, participants in the most deprived group adherent to unfavorable lifestyle had the highest risk of mental health outcomes. These results suggest that adherence to a broad combination of healthy lifestyle factors was associated with a significantly reduced risk of mental health conditions during the COVID-19 pandemic. Lifestyle factors, in conjunction with socioeconomic deprivation, independently contribute to the risk of mental health issues. Although further research is needed to assess causality, the current findings support public health strategies and individual-level interventions that provide enhanced support in areas of deprivation and target multiple lifestyle factors to reduce health inequalities and promote mental well-being during the ongoing COVID-19 pandemic.

## Introduction

Since the beginning of the COVID-19 pandemic, a range of public health interventions including social distancing, and lockdown (e.g., home confinement and isolation), have been urgently adopted by governments to contain the spread of the new coronavirus. The COVID-19 pandemic and related restrictions may potentially have negative psychological and social effects, such as financial insecurity, boredom, frustration, feeling a burden, loneliness, and fear, which are established risk factors for mental health outcomes including depression, anxiety, suicide, and self-harm [1]. These adverse effects are more evident in vulnerable population such as older adults and those who were socioeconomically deprived [2], further exacerbating physical and mental health inequalities within populations. Although the high levels of psychological symptoms in the early stage of the outbreak may partially decrease over time, emotionally vulnerable groups have remained at risk in the longer term [3]. Notably, the excess mental health burden related to the COVID-19 is further compounded by the long-term psychiatric sequelae of the SARS-CoV-2 infection [4]. Several population-level interventions were employed by policy makers to mitigate the adverse impact of pandemic and promote wellbeing [2], however, the contribution of individual-level interventions, such as modifiable lifestyle factors, to mental well-being remains unclear in the context of COVID-19 pandemic [5].

Modifiable lifestyle factors, including body mass index, smoking, alcohol consumption, physical activity, sleep duration, and diet, have been identified to be associated with mental disorders such as depression and anxiety [6], as well as cardiovascular disease [7] and all-cause mortality [8] in the non-COVID-19 setting. Previous studies also suggested that single pre-pandemic lifestyle factor such as physical activity or diet was associated with lower risk of psychological distress during the COVID-19 pandemic and lockdown [9, 10]. However, the association between combinations of lifestyle factors, which are known to interact synergistically [11], and risk of mental health outcomes in the pandemic setting has not been established. In addition, despite the potentially disproportionate impact of pandemic on socioeconomically disadvantaged populations, no studies have specifically examined how socioeconomic deprivation may modify the effectiveness of composite lifestyle factors in alleviating mental health conditions associated with COVID-19. Understanding these associations could help policy makers to target interventions at both the individual and population level to promote mental well-being, particularly among vulnerable groups in the current and future pandemic conditions.

Based on a nationally representative community-based cohort, we aim to assess the association between a combination of pre-pandemic lifestyle factors, including both emerging and traditional factors, and mental health conditions (depression, anxiety, and personal well-being) during the COVID-19 pandemic. In addition, we aim to examine the contribution of socioeconomic deprivation, as characterized by individual-level (income, wealth, and education) and group-level factors (Index of Multiple Deprivation), to the association between lifestyle factors and mental health conditions.

## Methods

### Study population

We used data from participants of the English Longitudinal Study of Ageing (ELSA), a longitudinal cohort that recruited a representative sample of adults aged 50 years and older living in private households in England, as detailed elsewhere [12]. The first wave of data collection took place on March 1, 2002, with subsequent longitudinal assessments every 2 years (wave 2 to wave 9) to measure changes in the health, economic and social circumstances using face-to-face interviews and self-administered questionnaires, and additional nurse visits every 4 years. The original sample based on Health Survey for England (HSE) included 11,391 participants, and there were further refreshment samples based on HSE at several waves (waves 3, 4, 6, 7 and 9) with different age criteria to correct for the age profile as the original sample aged. As certain lifestyle factors such as smoking and physical activity may have changed over a long period of time (e.g., a decade), the current study used the most recent data predated the COVID-19 pandemic (wave 9, 2018–19), and two waves of the ELSA COVID-19 substudy (COVID wave 1 and wave 2, collected in June/July and November/December 2020; 94% longitudinal response rate). Ethical approval was obtained from the National Research and Ethics Committee.

Analyses of this study are based on data from individuals who participated in both COVID-19 surveys with available information in wave 9 survey. In the two COVID-19 waves, participants were asked about the self-isolation (defined as not leaving home for any reason) and stay-at-home (defined as only leaving home for very limited purposes) conditions in April 2020 (early-stage of the outbreak), June/July and November/December 2020 (middle to late stage of the pandemic). The first and second national lockdown was enforced during the survey period [13], and those aged ≥70 years were considered clinically vulnerable and suggested by the UK Government to stay at home and shield [14]. Participants (381, 6.8%) who were not in self-isolation and did not stay at home at any of the three time points throughout the period were excluded.

### Socioeconomic characteristics

Socioeconomic deprivation was characterized by individual factors such as income, wealth, and education, and by group-level factor such as the Index of Multiple Deprivation (IMD). Education level was categorized as low (below secondary), middle and high (university or above), according to the International Standard Classification of Education [15]. Income was calculated from paid work, state benefits, pensions and assets [12]. Wealth was derived from net financial wealth that is gross financial wealth (e.g., property, possessions, housing, investments, savings) minus financial debt [12]. The IMD, encompassing domains such as crime, education, employment, health, housing, income, and living environment, served as the official measure of relative deprivation in England, representing the socio-economic status of individuals and communities [16, 17]. These factors were then categorized into low (lowest quintile), intermediate (quintiles 2 to 4), and high (highest quintile) groups to characterize socioeconomic disparities across different levels. In the main analyses, deprivation characterized by IMD was reported, which provided a comprehensive assessment of various socioeconomic characteristics. Individual socioeconomic factors, such as income and education, were used in the sensitivity analyses.

### Healthy lifestyles

We defined a composite healthy lifestyle score including 7 modifiable healthy lifestyle factors based on previous evidence and UK national health service guidelines [7, 18–20]: BMI, smoking status, alcohol consumption, physical activity, sedentary time, sleep duration, and fruit and vegetable intake. If available, we used UK national guidelines to generate healthy and unhealthy categories for each lifestyle factor [18]. One point was assigned for each unhealthy lifestyle category, including unhealthy body weight (BMI < 18.5 or ≥25), current smoker, high alcohol intake (daily or almost daily), moderate or vigorous physical activity less than once per week, <7 or >9 h of sleep per day, ≥7 h of sedentary time per day, and <5 portions of fruit and vegetable per day. Individuals’ scores were summed to create an unweighted score, and then classed as favorable (score 0–1), intermediate (score 2–3), or unfavorable (score 4–7) lifestyle category. Detailed definition of lifestyle category is provided in Supplementary Table 1. Distributions of the lifestyle score and lifestyle categories are shown in Supplementary Table 2.

### Mental health outcomes

The mental health outcomes in this analysis were depressive symptoms, anxiety and personal well-being. Depressive symptoms were measured by an abbreviated eight-item version of the validated Center for Epidemiologic Studies Depression Scale (CES-D 8) [21]. A score of ≥4 was used to define participants of elevated depressive symptoms [22]. Anxiety was measured by the seven-item Generalized Anxiety Disorder (GAD-7) scale [23], using a threshold score of ten to define clinically significant symptoms [24]. Although the results do not necessarily represent clinical diagnoses, the CES-D and GAD are validated scales that used in large-scale population-based studies to measure symptoms of depression and anxiety [21, 23]. Personal well-being was assessed by the four-item Office for National Statistics (ONS) well-being (ONS-4) scale that capture three types of well-being: evaluative, eudemonic and affective experience [25]. A score of ≤4 was used to define participants of low personal well-being [25].

### Covariates

Models were adjusted for a series of pre-pandemic covariates measured at baseline (wave 9), including age, sex, ethnicity, marital status, employment, disability, education, income, wealth, comorbidities and related conditions (chronic lung disease, asthma, arthritis, osteoporosis, cancer, Parkinson’s disease, dementia, hypertension, diabetes), and pre-pandemic mental health conditions (including history of psychiatric disorders, anxiety measured by ONS anxiety scale, depressive symptoms, and personal well-being) where applicable. In the sensitivity analyses, we additionally adjusted for pre-pandemic loneliness and social isolation that are risk factors for mental health outcomes and are anticipated direct consequences of pandemic and associated social and physical distancing. Loneliness was measured by the UCLA 3-item Loneliness Scale [26], and social isolation was measured by a composite score as in previous ELSA studies [27], in which one point was allocated for each of the following: not being married or cohabiting; having less than monthly contact with each child, other members of the family, and friends (one point for each); and not being a member of organizations, such as religious groups or social groups.

### Statistical analysis

We assess the associations between lifestyle factors, socioeconomic deprivation, and subsequent mental health conditions using Cox proportional hazards model, with study wave as the timescale that was adjusted for covariates.

Proportional hazard assumptions were checked based on Schoenfeld residuals and were satisfied. First, we separately assessed the association of composite lifestyle score (0–7; continuous variable) and lifestyle category (favorable, intermediate, and unfavorable) with mental health conditions, with adjusted for the above covariates and additionally for socioeconomic characteristics (education, income, and wealth). Second, we examined whether socioeconomic deprivation modified the association of lifestyle factors and mental health conditions. Multiplicative interactions between lifestyle category and socioeconomic deprivation characterized by group-level factor (IMD) were tested, with *P* values reported. We quantified the association between lifestyle category and mental health conditions across groups of socioeconomic deprivation, with the favorable lifestyle category as the reference group. We also estimated the combined effect of lifestyle and socioeconomic deprivation using nine ordinal categories, with participants in the least deprived group who were in the favorable lifestyle category as the reference group. The hazard ratio (HR) for trend per one increment change in lifestyle category was calculated. All models were adjusted for confounders including age, sex, ethnicity, marital status, employment, disability, comorbidities and related conditions. To account for potential reverse causality, pre-pandemic mental health was also adjusted, including history of psychiatric disorders, symptoms of depression and anxiety, and personal well-being.

Several sensitivity analyses were conducted to assess the robustness of the main analyses. First, in addition to the IMD as the primary socioeconomic characteristics, individual-level factors including education, income, and wealth were used to examine the contribution of socioeconomic deprivation. Second, we assessed the association between individual lifestyle factors (e.g., past or never smoker vs current smoker) and mental health conditions. Third, we run analyses after excluding participants with history of mental disorders. Forth, we additionally adjusted for pre-pandemic level of loneliness and social isolation that may be associated with both exposures and outcomes or mediate the association between lifestyle and mental health conditions during the COVID-19 pandemic. Finally,

All analyses were performed using SAS version 9.4 (SAS Institute) and R version 4.2.2 (R Foundation), and all statistical tests were two-sided, with *p* < 0.05 considered significant.

## Results

### Population characteristics

Of the 5594 participants assessed in two waves of the ELSA COVID-19 substudy, 5213 (93.2%) experienced self-isolation or shielding during the national lockdown. Of these, 5049 (96.8%) had available pre-pandemic baseline data on lifestyle factors, measures of socioeconomic deprivation, and covariates. Table 1 shows the baseline characteristics of 5049 eligible participants by lifestyle category. Overall, the mean (SD) age was 68.1 (10.9) years (range 50–90 years), of whom 57.2% were female and 96.1% were White. 2101 (41.6%) participants were in the favorable lifestyle category, 2468 (48.9%) in the intermediate lifestyle category, and 480 (9.5%) in the unfavorable lifestyle category. In general, participants in the unfavorable lifestyle category were more likely to be older, male, and socioeconomically deprived (e.g., lower income, wealth, and education), and to have less individual healthy lifestyle (e.g., higher BMI), more comorbidities, and worse pre-pandemic mental health conditions, and higher levels of loneliness and social isolation. The prevalence was 26.6% for symptom of depression, 9.2% for symptom of anxiety, and 8.1% for low personal well-being during the COVID-19 pandemic and lockdown.

**Table 1:** Baseline characteristics by lifestyle category.

	Total (*n* = 5049)	Lifestyle category^a^		
		Favorable (*n* = 2101)	Intermediate (*n* = 2468)	Unfavorable (*n* = 480)
Age, mean (SD), *y*	68.1 (10.9)	66.5 (11.5)	69.0 (10.1)	69.7 (8.5)
Sex (%)				
Male	2163 (42.8)	793 (37.7)	1143 (46.3)	227 (47.3)
Female	2886 (57.2)	1308 (62.3)	1325 (53.7)	253 (52.7)
Ethnicity (%)				
White	4853 (96.1)	1988 (94.6)	2395 (97.0)	470 (97.9)
Non-white	194 (3.8)	111 (5.3)	73 (3.0)	10 (2.1)
Employment				
Retired	3343 (66.2)	1285 (61.2)	1709 (69.2)	349 (72.7)
Employed	1291 (25.6)	648 (30.8)	565 (22.9)	78 (16.2)
Unemployed, sick, disabled, and others	415 (8.2)	168 (8.0)	194 (7.9)	53 (11.0)
Marriage				
Single	360 (7.1)	162 (7.7)	163 (6.6)	35 (7.3)
Married or remarried	3322 (63.2)	1407 (67.0)	1633 (66.2)	282 (58.8)
Separated	67 (1.3)	26 (1.2)	37 (1.5)	4 (0.8)
Divorced	629 (12.5)	229 (10.9)	315 (12.8)	85 (17.7)
Widowed	670 (13.3)	276 (13.1)	320 (13.0)	74 (15.4)
Educational attainment (%)				
Low (below secondary)	1554 (30.8)	561 (26.7)	791 (32.1)	202 (42.1)
Middle	2245 (44.5)	966 (46.0)	1094 (44.3)	185 (38.5)
High (university or above)	1250 (24.8)	574 (27.3)	583 (23.6)	93 (19.4)
Income per month, quintile (%)				
1 (< £612)	801 (15.9)	331 (15.8)	378 (15.3)	92 (19.2)
2 (£612–£824)	984 (19.5)	389 (18.5)	498 (20.2)	97 (20.2)
3 (£824–£1153)	1031 (20.4)	432 (20.6)	485 (19.7)	114 (23.8)
4 (£1153–£1431)	1081 (21.4)	442 (21.0)	543 (22.0)	96 (20.0)
5 ( > £1,431)	1060 (21.0)	461 (21.9)	526 (21.3)	73 (15.2)
Wealth, quintile (%)				
1 (< £7.2k)	622 (12.3)	199 (9.5)	322 (13.0)	101 (21.0)
2 (£7.2K–£21.6 K)	815 (16.1)	360 (17.1)	370 (15.0)	85 (17.7)
3 (£21.6K–£37.6 K)	1100 (21.8)	455 (21.7)	542 (22.0)	103 (21.5)
4 (£37.6K–£64.6 K)	1188 (23.5)	496 (23.6)	592 (24.0)	100 (20.8)
5 ( > £64.6 K)	1232 (24.4)	545 (25.9)	604 (24.5)	83 (17.3)
Index of multiple deprivation, quintile (%)				
1 (most deprived)	1357 (26.9)	579 (27.6)	668 (27.1)	110 (22.9)
2–4	3167 (62.7)	1336 (63.6)	1539 (62.4)	292 (60.8)
5 (least deprived)	485 (9.6)	171 (8.1)	240 (9.7)	74 (15.4)
BMI, mean (SD), kg/m^2^	27.9 (5.2)	25.8 (4.5)	28.6 (5.1)	30.5 (5.6)
Underweight (<18.5)	44 (0.9)	13 (0.6)	28 (1.1)	3 (0.6)
Normal weight (18.5–24.9)	1080 (21.4)	681 (32.4)	376 (15.2)	23 (4.8)
Overweight (25.0–29.9)	2765 (54.8)	1170 (55.7)	1338 (54.2)	257 (53.5)
Obese (≥30.0)	1099 (21.8)	211 (10.0)	692 (28.0)	196 (40.8)
Time spent sitting, mean (SD), h	4.8 (4.1)	3.3 (3.7)	5.4 (4.0)	7.9 (4.2)
Sleep duration, mean (SD), h	8.7 (3.0)	8.1 (1.6)	8.8 (3.1)	10.4 (5.0)
Healthy lifestyle factors, %				
Healthy body weight (BMI, 18.5–24.9)	1080 (21.4)	681 (32.4)	376 (15.2)	23 (4.8)
No current smoking (past or never smoker)	4668 (92.5)	2042 (97.2)	2263 (91.7)	363 (75.6)
Moderate alcohol consumption (≤4 times/w)	4165 (82.5)	1985 (94.5)	1905 (77.2)	275 (57.3)
Regular physical activity (moderate or vigorous activity ≥1/w)	4063 (80.5)	1970 (93.8)	1879 (76.1)	214 (44.6)
Less sedentary behavior (<7 h/d)	3676 (72.8)	1933 (92.0)	1604 (65.0)	139 (29.0)
Adequate sleep duration (7–9 h/d)	3816 (75.6)	1949 (92.8)	1726 (69.9)	141 (29.4)
Adequate fruit and vegetable intake (≥5 portion/d)	3281 (65.0)	1885 (89.7)	1269 (51.4)	127 (26.5)
Comorbidity				
Chronic lung disease	189 (3.7)	53 (2.5)	101 (4.1)	35 (7.3)
Asthma	466 (9.2)	158 (7.5)	251 (10.2)	57 (11.9)
Arthritis	1856 (36.8)	634 (30.2)	983 (39.8)	239 (49.8)
Osteoporosis	355 (7.0)	133 (6.3)	179 (7.3)	43 (9.0)
Cancer	236 (4.7)	96 (4.6)	111 (4.5)	29 (6.0)
Parkinson’s disease	22 (0.4)	3 (0.1)	18 (0.7)	1 (0.2)
Dementia	18 (0.4)	1 (0.0)	11 (0.4)	6 (1.2)
Hypertension	1596 (31.6)	506 (24.1)	892 (36.1)	198 (41.2)
Diabetes	426 (8.4)	112 (5.3)	244 (9.9)	70 (14.6)
Pre-pandemic mental health related conditions, %				
History of diagnosed mental disorders	308 (6.1)	98 (4.7)	168 (6.8)	42 (8.8)
Symptoms of depression (CES-D ≥ 4)	522 (10.3)	161 (7.7)	285 (11.5)	76 (15.8)
Low personal well-being (ONS ≤ 4)	712 (14.1)	392 (18.7)	257 (10.4)	63 (13.1)
Loneliness (UCLA scale), mean (SD)	3.2 (3.3)	2.7 (3.5)	3.5 (3.2)	4.1 (2.3)
Social isolation score, mean (SD)^b^	1.4 (1.1)	1.3 (1.0)	1.4 (1.1)	1.6 (1.1)

Data are mean (SD) or *n* (%) for continuous and categorical variables, as appropriate.

*W* week, *D* day, *Y* year, *K* thousand, *SD* standard deviation.

^a^Based on composite lifestyle score, participants were categorized as favorable (score 0–2), intermediate (score 3–5), or unfavorable (score 6–9) lifestyle.

^b^Social isolation was defined by a composite score based on multiple questions on social activity.

### Healthy lifestyle and mental health

**Fig. 1:**
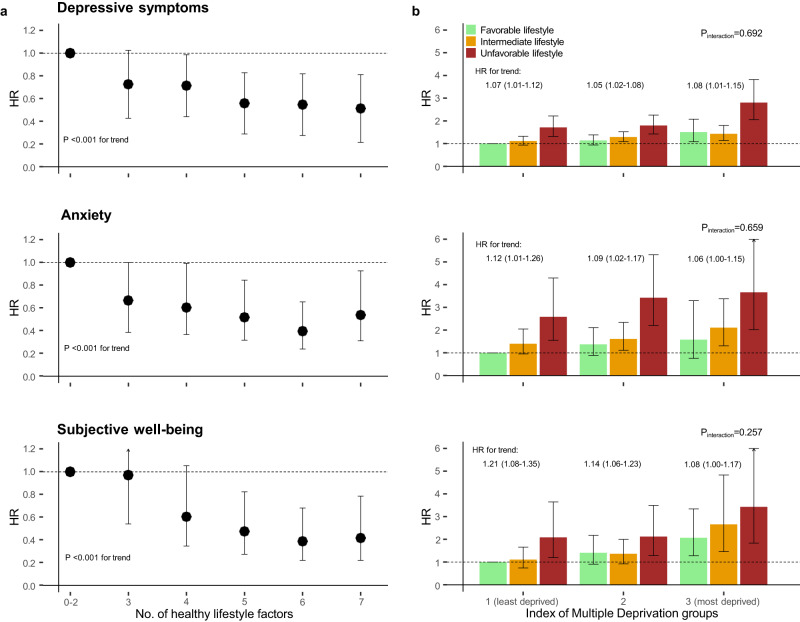
Association between lifestyle category, socioeconomic deprivation group, and mental health conditions. **a** Number of healthy lifestyle factors prior to the pandemic and risk of mental health conditions during the COVID-19 pandemic. The seven healthy lifestyle factors include healthy body weight (BMI, 18.5–24.9), no currently smoking, moderate alcohol intake (≤4 times/wk), at least 1 time/wk of moderate to vigorous physical activity, and adequate sleep (7–9 h/d), and adequate intake of fruit and vegetable consumption (≥5 portion/d). Model was adjusted for age, sex, ethnicity, marital status, employment, education, income, wealth, comorbidities, and pre-pandemic mental health conditions. **b** Lifestyle category, socioeconomic deprivation group and risk of mental health conditions during the COVID-19 pandemic. The Model was adjusted for age, sex, ethnicity, marital status, comorbidities, and pre-pandemic mental health conditions. Error bars indicate 95% CIs. HR: hazard ratio. HR for trend indicates the change in HR by one lifestyle* IMD category change towards unfavorable within each IMD category. Participants in the least deprived group who were in the favorable lifestyle category were defined as the reference group.

The association between lifestyle category and mental conditions are shown in Table 2. Compared with the favorable lifestyle category, participants in the intermediate and unfavorable lifestyle categories were at increased risk of all mental health outcomes. One increment change towards the unfavorable healthy category was associated with a HR for trend of 1.17 (95% CI 1.09–1.26) for depression, 1.23 (1.07–1.42) for anxiety, and 1.39 (1.20–1.61) for low well-being. There was a clear and significant trend of lower risk for all mental health outcomes with increasing number of healthy lifestyle factors (Fig. 1; *P* < .001 for trend). Compared with those who adhered to only 0–2 healthy lifestyle factors, those having seven factors had a 48% lower risk of depression (HR, 0.52; 95% CI, 0.39–0.69), a 42% lower risk of anxiety (0.58, 0.35–0.98), and a 58% lower risk of low well-being (0.42, 0.23–0.75). One increment change in lifestyle score was associated with a HR for trend of 1.11 (95% CI 1.07–1.16) for depression, 1.11 (1.03–1.19) for anxiety, and 1.20 (1.10–1.30) for low well-being. In sensitivity analyses assessing each lifestyle factor separately, BMI, smoking, physical activity, and sleep duration were independently associated with risk of mental health outcomes after mutually adjusted for all lifestyle factors and covariates (Table 3).

**Table 2:** Association between lifestyle category and mental health conditions.

	Total (*n* = 5049)	Favorable lifestyle (*n* = 2101)		Intermediate lifestyle (*n* = 2468)		Unfavorable lifestyle (*n* = 480)		HR for trend (95% CI)
		Events	HR	Events	HR (95% CI)	Events	HR (95% CI)	
Depression	1342 (26.6%)	514 (24.5%)	1 (ref)	657 (26.6%)	1.14 (1.04–1.26)	171 (35.6%)	1.41 (1.21–1.65)	1.17 (1.09–1.26)
Anxiety	466 (9.2%)	171 (8.1%)	1 (ref)	236 (9.6%)	1.23 (1.01–1.50)	59 (12.3%)	1.51 (1.12–2.05)	1.23 (1.07–1.42)
Well-being	408 (8.1%)	141 (6.7%)	1 (ref)	203 (8.2%)	1.25 (1.00–1.56)	64 (13.3%)	2.04 (1.51–2.75)	1.39 (1.20–1.61)

The favorable lifestyle category was used as reference category. HR for trend indicates the change in HR by one lifestyle category change towards unfavorable. The Model was adjusted for age, sex, ethnicity, marital status, employment, education, income, wealth, comorbidities, and pre-pandemic mental health conditions. *HR* hazard ratio.

**Table 3:** Association between individual lifestyle factor and mental health conditions.

Lifestyle factors	Depression		Anxiety		Well-being	
	Events	HR (95% CI)	Events	HR (95% CI)	Events	HR (95% CI)
BMI						
<18.5 (underweight)	26 (59.1%)	1.87 (1.25–2.80)	7 (15.9%)	1.63 (0.75–3.57)	3 (6.8%)	0.88 (0.28–2.79)
18.5–24.9 (ref)	351 (32.5%)	1 (ref)	79 (7.3%)	1 (ref)	74 (6.8%)	1 (ref)
25–29.9 (overweight)	539 (32.8%)	1.04 (0.91–1.20)	101 (6.2%)	0.83(0.61–1.11)	131 (8.0%)	1.26 (0.94–1.68)
≥30 (obese)	497 (44.0%)	1.31 (1.14–1.51)	134 (11.9%)	1.33 (1.00–1.78)	109 (9.6%)	1.40 (1.03–1.91)
Smoking						
Never or past	1707 (36.6%)	1 (ref)	400 (8.6%)	1 (ref)	342 (7.4%)	1 (ref)
Current	197 (51.7%)	1.38 (1.18–1.61)	66 (17.3%)	1.67 (1.18–2.35)	62 (16.3%)	2.08 (1.46–2.96)
Alcohol consumption						
≤4 times/week	1618 (38.8%)	1 (ref)	403 (9.7%)	1 (ref)	341 (8.2%)	1 (ref)
Daily or almost daily	286 (32.4%)	0.89 (0.77–1.03)	63 (7.1%)	0.80 (0.58–1.12)	67 (7.6%)	1.09 (0.81–1.46)
Physical activity						
≥1 time/week	1376 (33.9%)	0.64 (0.67–0.72)	322 (7.9%)	0.69 (0.54–0.88)	279 (6.9%)	0.67 (0.61–0.86)
<1 time/week	528 (53.5%)	1 (ref)	144 (14.6%)	1 (ref)	129 (13.1%)	1 (ref)
Sedentary behavior						
<7 h/d	1351 (36.8%)	1 (ref)	335 (9.1%)	1 (ref)	281 (7.6%)	1 (ref)
≥7 h/d	553 (40.3%)	1.08 (0.96–1.22)	131 (9.5%)	1.05 (0.82–1.34)	127 (9.2%)	1.18 (0.93–1.51)
Sleep duration						
7–9 h/d	1351 (35.4%)	0.80 (0.71–0.90)	314 (8.2%)	0.69 (0.54–0.87)	290 (7.6%)	0.94 (0.73–1.21)
<7 or >9 h/d	553 (44.8%)	1 (ref)	152 (12.3%)	1 (ref)	118 (9.6%)	1 (ref)
Fruit and vegetable intake						
≥5 portion/day	1119 (34.1%)	0.88 (0.79–0.98)	263 (8.0%)	0.91 (0.73–1.15)	233 (7.1%)	0.88 (0.70–1.12)
<5 portion/day	701 (39.6%)	1 (ref)	175 (9.9%)	1 (ref)	152 (8.6%)	1 (ref)

Model was adjusted for age, sex, ethnicity, marital status, comorbidities, pre-pandemic mental health conditions, and other lifestyle factors included in the table.

*HR* hazard ratio, *BMI* body mass index.

### Socioeconomic deprivation and mental health

Socioeconomic deprivation characterized by IMD was associated with a higher risk of all mental health outcomes (Table S3). One increment in the deprivation category was associated with a HR for trend of 1.25 (95% CI 1.15–1.35) for depression, 1.57 (1.34–1.84) for anxiety, and 1.22 (1.03–1.44) for low well-being. In sensitivity analyses that deprivation was characterized by individual-level socioeconomic factors including education, income, and wealth, deprivation was consistently associated with higher risk of all mental health outcomes (Tables S4–S6).

### Healthy lifestyle, socioeconomic deprivation and mental health

When stratified by socioeconomic deprivation group as characterized by IMD, participants in the unfavorable lifestyle category were at increased risk of depression, anxiety, and low well-being compared with those in the favorable lifestyle category across deprivation groups (Table 4). There was also significant trend of increasing risk of mental health outcomes per one increment in lifestyle category across deprivation groups (Table 4).

**Table 4:** Association between lifestyle category and mental health conditions by socioeconomic deprivation group.

	Total (*n* = 5049)	Favorable lifestyle (*n* = 2101)		Intermediate lifestyle (*n* = 2468)		Unfavorable lifestyle (*n* = 480)		HR for trend (95% CI)
		Events	HR (95% CI)	Events	HR (95% CI)	Events	HR (95% CI)	
Depression								
Least deprived	299/1357 (22.0%)	112 (19.3%)	1 (ref)	151 (22.6%)	1.15 (0.94–1.40)	36 (32.7%)	1.50 (1.09–2.01)	1.20 (1.03–1.39)
Intermediate	826/3167 (26.1%)	334 (25.0%)	1 (ref)	399 (25.9%)	1.15 (0.87–1.31)	93 (31.8%)	1.28 (1.05–1.57)	1.14 (1.04–1.25)
Most deprived	203/485 (41.9%)	64 (37.4%)	1 (ref)	98 (40.8%)	1.06 (0.80–1.41)	41 (55.4%)	1.67 (1.18–2.39)	1.26 (1.05–1.52)
Anxiety								
Least deprived	94/1357 (6.9%)	35 (6.0%)	1 (ref)	50 (7.5%)	1.41 (0.91–2.19)	9 (8.2%)	1.64 (0.78–3.43)	1.33 (0.96–1.83)
Intermediate	280/3167 (8.8%)	109 (8.2%)	1 (ref)	137 (8.9%)	1.16 (0.90–1.49)	34 (11.6%)	1.52 (1.03–2.25)	1.21 (1.01–1.45)
Most deprived	88/485 (18.1%)	26 (15.2%)	1 (ref)	46 (19.2%)	1.37 (0.84–2.23)	16 (21.6%)	1.54 (0.81–2.89)	1.26 (0.93–1.70)
Well-being								
Least deprived	102/1357 (7.5%)	33 (5.7%)	1 (ref)	53 (7.9%)	1.40 (0.90–2.17)	16 (14.5%)	2.62 (1.43–4.78)	1.57 (1.16–2.13)
Intermediate	238/3167 (7.5%)	85 (6.4%)	1 (ref)	119 (7.7%)	1.22 (0.92–1.61)	34 (11.6%)	1.84 (1.23–2.74)	1.32 (1.08–1.61)
Most deprived	63/485 (13.0%)	20 (11.7%)	1 (ref)	29 (12.1%)	1.08 (0.61–1.92)	14 (18.9%)	1.72 (0.85–3.46)	1.29 (0.90–1.85)

The favorable lifestyle category for each group of deprivation was used as the reference category. HR for trend indicates the change in HR by one lifestyle category change towards unfavorable. Model was adjusted for age, sex, ethnicity, marital status, comorbidities, and pre-pandemic mental health conditions and levels of loneliness and social isolation.

When all groups were compared with a single reference group including least deprived participants with favorable lifestyle, there was a dose-response increment for all mental health outcomes across most lifestyle categories and deprivation groups (Fig. 1). The highest risks were observed among deprived participants with unfavorable lifestyle. Compared with those in the least deprived group with favorable lifestyle, participants in the most deprived group adherent to unfavorable lifestyle had increased risk of depression (HR, 2.80; 95% CI, 2.05–3.82), anxiety (3.66, 2.02–6.64), and low well-being (3.43, 1.83–6.42). One increment in the lifestyle x deprivation category was associated with higher risk of depression (HR for trend, 1.07; 95% CI, 1.05–1.10), anxiety (1.11, 1.07–1.16), and low well-being (1.13, 1.07–1.18). There were no significant interactions between lifestyle factors and socioeconomic deprivation for any of the mental health outcomes (Fig. 1). Similar HRs for trend for one increment change towards unfavorable lifestyle were observed in each deprivation group. The HR for trend per one increment change in lifestyle within the least deprived versus most deprived groups was 1.07 (1.01–1.12) vs 1.08 (1.01–1.15) for depression, 1.12 (1.01–1.26) vs 1.06 (1.00–1.15) for anxiety, and 1.21 (1.08–1.35) vs 1.08 (1.00–1.17) for low well-being.

In sensitivity analyses, the association between lifestyle, deprivation characterized by individual-level socioeconomic factors, and mental health outcomes were broadly consistently with the main analyses (Tables S7–S9). Participants with the lowest income, wealth, or education level in the unfavorable lifestyle category had the highest HR for all mental health outcomes. The interaction patterns between lifestyle and individual-level socioeconomic factors were similarly to the pattern associated the IMD (Fig. S1). Sensitivity analyses excluding those history of mental disorders or analyses further adjusted for pre-pandemic levels of loneliness and social isolation showed similar pattern between lifestyle, deprivation as characterized by IMD, and mental health outcomes (Fig. S2).

## Discussion

In this longitudinal study of a nationally representative population in England, our findings suggest that a broad combination of pre-pandemic lifestyle factors, including BMI, smoking, alcohol consumption, physical activity, sedentary behavior, sleep duration, and fruit or vegetable intake, were associated with risk of mental health conditions such as depression, anxiety, and low personal well-being during the COVID-19 pandemic regardless of deprivation level. Dose-response protective associations of the number of healthy lifestyle factors with risk of mental health conditions were observed, after adjusting for sociodemographic factors and other pre-pandemic conditions. In addition, we found lifestyle factors and socioeconomic deprivation were independently associated with risk of mental health conditions, with no significant interaction observed. Unfavorable lifestyle and deprivation have a log-additive association with the risk of mental health conditions, and the relative association of lifestyle factors are similar across deprivation groups.

Few studies have investigated the association between modifiable lifestyle factors predates the pandemic and mental health conditions during the pandemic and lockdown setting. It is well-established that the COVID-19 pandemic and associated restrictions have had negative psychological effects, especially on vulnerable groups such as older adults, socioeconomically disadvantaged individuals, and those with mental disorders [1, 2]. However, previous evidence was primarily focused on population-level recommendations for promoting mental health and well-being during the pandemic, specific interventions targeting on individuals were lacking [1, 5]. This gap in individual-level recommendations could be due to a lack of sufficient evidence in this area. Previous studies in the non-COVID-19 setting suggested that adherence to a healthy lifestyle were associated with lower risk of noncommunicable diseases such as cardiovascular diseases and mental disorders [28], communicable diseases such as COVID-19, and all-cause mortality. However, uncertainties persist regarding whether these associations remain in the COVID-19 setting, where stressful and disruptive societal changes during the pandemic and lockdown could potentially lead to changes in behaviors and exacerbate mental health issues. This study identified that pre-pandemic composite lifestyle scores or lifestyle categories were associated with mental health conditions in a dose-response manner independent of pre-existing conditions and other potential confounding factors.

In the analyses mutually adjusted for all individual lifestyle factors and covariates, BMI, smoking, physical activity, and sleep duration were likely to be the main driver of the association between lifestyle and mental health. These findings align with evidence from mendelian randomization studies, which indicate that individual lifestyle factors such as BMI [29], physical activity [30], smoking [31], and sleep traits [32], potentially have causal effects on the risk of mental disorders and may be an effective prevention strategy. Our findings further highlight the potential benefits of maintaining a healthy lifestyle for promoting mental health during the pandemic and lockdown period, particularly considering the expected increase in psychological symptoms during this time.

Socioeconomic deprivation had severe negative impacts on mental health and well-being through social stresses, stigma and trauma [33], which was further exacerbated during the COVID-19 pandemic and lockdown [2]. Deprivation and related factors, such as psychosocial stress, can also lead to disproportionate lifestyle harm, potentially through extremes of unhealthy lifestyle factors and reduced access to health services [34]. Previous study in the UK Biobank suggested that deprivation modified the association between composite lifestyle factors and mortality but not incidence of cardiovascular diseases [34], while the contribution of deprivation to the association with mental health, especially during the pandemic, remains unclear. In this study, no significant interaction between lifestyle and socioeconomic deprivation was observed, suggesting that these two factors may independently affect mental health during the pandemic setting. In addition, the potential benefit of adhering to healthy lifestyle was similar across deprivation groups.

In light of our findings and previous evidence, we make recommendations at individual and population level that may help mitigate the excess burden of mental health during the ongoing pandemic and promote wellbeing. First, if the observed associations were causal, individual-level lifestyle interventions should be informed, accompanied by effective and timely mental health and social support, regardless of the deprivation level. Second, expanding the scope of targeted lifestyle factors, such as maintaining a healthy body weight, quitting smoking, engaging in regular physical activity, and ensuring sufficient sleep duration, may offer greater benefits for mental well-being. Third, our findings provide further support for the global goal of reducing poverty, and the health policies for increased support in areas of deprivation. Addressing the socioeconomic deprivation and improving wider combinations of lifestyle factors are both important to reduce the expanding mental health inequalities during the COVID-19 pandemic. Behavioral lifestyle changes should also be encouraged and integrated into the current public health and individual-level interventions [1, 5] to empower mental well-being.

To our knowledge, this is the first longitudinal study to assess the association of a comprehensive set of lifestyle factors with the risk of mental health conditions during the COVID-19 pandemic. The strength of the current study includes the use of a nationally representative population and a prospective design in which a range of well-defined lifestyle and sociodemographic factors were assessed prior to the pandemic using validated instruments. Notably, the assessment of lifestyle factors was based on the most recent survey conducted prior to the COVID-19 pandemic to minimize the potential influence of changes in behavior over the long term, which could have occurred if an earlier survey had been used. Moreover, our study additionally benefited from a more comprehensive evaluation of socioeconomic status incorporating both individual and area-based factors compared to the limited approaches utilized in previous research. The wealth was calculated based on multiple individual financial components, rather than simple classification of assets. Our study contributes valuable population-based evidence regarding the association between healthy lifestyle, socioeconomic deprivation and mental health during the pandemic.

However, several limitations should be noted. First, although ELSA is a nationally representative community-based cohort, participants were aged ≥50 years and 96% were white, which may limit the generalizability of our findings to other younger populations of other ethnicities. Second, although a series of pre-pandemic covariates were accounted for and participants with history of mental disorders were excluded in the sensitivity analyses, residual confounding and reverse causality cannot be ruled out in this observational study. Further study with alternative study design such as mendelian randomization are warranted to assess whether the identified associations are causal. Third, although lifestyle factors were measured in the most recent survey predated the COVID-19 pandemic, it is likely that the pandemic and related restrictions could lead to changes in behaviors such as decreased physical activity and having a poor diet [35, 36]. However, this tends to make the risk estimates underestimate and thus lead to more conservative results. Forth, lifestyle factors, with the exception of BMI, were self-reported by participants, which may introduce healthy reporting bias wherein individuals may tend to overestimate or present a more positive picture of their lifestyle behaviors [37]. However, this potential bias may potentially lead to an underestimation of the observed associations and bias the true association towards null. Fifth, as with similar studies, it is important to recognize that while lifestyle factors are potentially modifiable, the beneficial effect of healthy lifestyle likely takes years to manifest in individuals and implementing lasting changes can be challenging especially for several populations such as older adults. Modification or restoration of lifestyle behavior through short-term interventions may not meaningfully improve mental well-being during the pandemic condition [35]. Finally, mental health conditions was measured during the pandemic and national lockdown. However, the association between individual lifestyles and mental disorders has been observed across diverse geographic regions and cultural backgrounds before the pandemic [32, 38], indicating the broad and generalizable nature of the relationship. The association between composite lifestyle factors, socioeconomic deprivation, and mental well-being observed in the current study is likely to extend beyond the COVID-19 pandemic and lockdown setting (e.g., general setting and future pandemic).

## Conclusion

This prospective cohort study reveals that adherence to a broad combination of healthy lifestyle factors was associated with a significantly reduced risk of mental health conditions during the COVID-19 pandemic. Lifestyle factors, in conjunction with socioeconomic deprivation, independently contribute to the risk of mental health issues, potentially exacerbating health inequalities. If these associations are causal, public health and individual-level interventions that provide enhanced support in areas of deprivation and target multiple lifestyle factors should be informed and implemented to reduce health inequalities and promote mental well-being during the current and future pandemic.

### Supplementary information

SUPPLEMENTAL MATERIAL

## Data Availability

The ELSA data used in the current study were available for apply through the UK Data Service (https://ukdataservice.ac.uk/).

## Code availability

The code generated and used to produce this manuscript is available from the corresponding authors on request.
