# Supplementary material for: Composite healthy lifestyle, socioeconomic deprivation, and mental well-being during the COVID-19 pandemic: a prospective analysis
Source: Mol Psychiatry. 2023 Dec 19;29(2):439–48. doi: 10.1038/s41380-023-02338-y (PMC11116094; doi:10.1038/s41380-023-02338-y)
Supplement: Supplementary file 1 — SUPPLEMENTAL MATERIAL [file 41380_2023_2338_MOESM1_ESM.docx]

**Supplemental Online Content**

**Composite healthy lifestyle, socioeconomic deprivation, and mental well-being during the COVID-19 pandemic: a prospective analysis**

| **Table of Contents** | **Page** |
| --- | --- |
| **Supplementary tables** |  |
| Table S1. Definition of healthy lifestyle factors in ELSA | **1** |
| Table S2. Distribution of lifestyle score and lifestyle categories | **2** |
| Table S3. Association between deprivation category characterized by IMD and mental health conditions | **3** |
| Table S4. Association between deprivation category characterized by income and mental health conditions | **4** |
| Table S5. Association between deprivation category characterized by wealth and mental health conditions | **5** |
| Table S6. Association between deprivation category characterized by education and mental health conditions | **6** |
| Table S7. Association between lifestyle category and mental health conditions by socioeconomic deprivation group characterized by income | **7** |
| Table S8. Association between lifestyle category and mental health conditions by socioeconomic deprivation group characterized by wealth | **8** |
| Table S9. Association between lifestyle category and mental health conditions by socioeconomic deprivation group characterized by education | **9** |
| **Supplementary Figures** |  |
| Fig. S1. Association between lifestyle category, socioeconomic deprivation group characterized by individual-level factors (income, wealth, and education), and mental health conditions | **10** |
| Fig. S2. Sensitivity analyses of the association between lifestyle category, socioeconomic deprivation, and mental health conditions. | **12** |

**Table S1. Definition of healthy lifestyle factors in ELSA**

| **Healthy lifestyle factors** | **Description** | **Healthy** | **Unhealthy** |
| --- | --- | --- | --- |
| **BMI** | Body mass index calculated as weight in kilograms divided by height in meters squared | BMI, 18.5-24.9 | BMI, <18.5 or ≥25 |
| **Smoking** | Current and past smoking status | Past or never smoker | Current smoker |
| **Alcohol consumption** | Current alcohol intake | ≤4 times/week | Daily or almost daily |
| **Physical activity** | Frequency of participation in vigorous, moderate and mild physical activities | Physically active (moderate or vigorous activity at least once per week) | Inactive  (less than once moderate or vigorous activity per week) |
| **Sedentary behavior** | Time spent sitting in a 24-hour period | <7h/d | ≥7h/d |
| **Sleep duration** | Average sleep duration in a 24-hour period | 7-9 h/d | <7 or >9h/d |
| **Fruit and vegetable intake** | Combined intake of fruit and vegetable per day, converted to portion/day | ≥5 portion/day | <5 portion/day |

One point was assigned for each unhealthy lifestyle category (incorporating unhealthy sleep duration and high sedentary time, smoking, excessive alcohol, low intake of fish

**Table S2. Distribution of lifestyle score and lifestyle categories**

| **Lifestyle category** | **Lifestyle score** | **No. of participants (n=5049)** | | **Percentage (%)** | |
| --- | --- | --- | --- | --- | --- |
| Favorable lifestyle | 0 | 664 | 2101 | 13.2 | 41.6 |
|  | 1 | 1437 |  | 28.5 |  |
| Intermediate lifestyle | 2 | 1472 | 2468 | 29.2 | 48.9 |
|  | 3 | 996 |  | 19.7 |  |
| Unfavorable lifestyle | 4 | 371 | 480 | 7.3 | 9.5 |
|  | 5 | 98 |  | 1.9 |  |
|  | 6 | 10 |  | 0.2 |  |
|  | 7 | 1 |  | 0.0 |  |

**Table S3. Association between deprivation category characterized by IMD and mental health conditions**

|  | **Total (n=5049)** | **Least deprived**  **(n=1357)** | | **Intermediate**  **(n=3167)** | | **Most deprived**  **(n=485)** | | **HR for trend (95% CI)** |
| --- | --- | --- | --- | --- | --- | --- | --- | --- |
|  |  | Events | HR | Events | HR (95% CI) | Events | HR (95% CI) |  |
| Depression | 1342 (26.6%) | 299 (22.0%) | 1 (ref) | 826 (26.1%) | 1.10 (1.00-1.23) | 203 (41.9%) | 1.66 (1.43-1.94) | 1.25 (1.15-1.35) |
| Anxiety | 466 (9.2%) | 94 (6.9%) | 1 (ref) | 280 (8.8%)） | 1.27 (1.01-1.61) | 88 (18.1%) | 2.50 (1.86-3.35) | 1.57 (1.34-1.84) |
| Well-being | 408 (8.1%) | 102 (7.5%) | 1 (ref) | 238 (7.5%) | 1.01 (0.78-1.24) | 63 (13.0%) | 1.63 (1.19-2.24) | 1.22 (1.03-1.44) |

Based on the IMD, participants were categorized into low (lowest quintile), intermediate (quintiles 2 to 4), and high (highest quintile) groups to characterize socioeconomic deprivation. Model was adjusted for age, sex, ethnicity, marital status, comorbidities, pre-pandemic mental health conditions, and lifestyle factors. HR for trend indicates the change in HR by one category increment in socioeconomic deprivation towards most deprived. HR=hazard ratio.

**Table S4. Association between deprivation category characterized by income and mental health conditions**

|  | **Total (n=5049)** | **Least deprived**  **(n=1060)** | | **Intermediate**  **(n=3096)** | | **Most deprived**  **(n=801)** | | **HR for trend (95% CI)** |
| --- | --- | --- | --- | --- | --- | --- | --- | --- |
|  |  | Events | HR | Events | HR (95% CI) | Events | HR (95% CI) |  |
| Depression | 1342 (26.6%) | 179 (16.9%) | 1 (ref) | 812 (26.2%) | 1.46 (1.28-1.66) | 325 (40.6%) | 2.05 (1.76-2.38) | 1.43 (1.32-1.54) |
| Anxiety | 466 (9.2%) | 56 (5.3%) | 1 (ref) | 276 (8.9%)） | 1.67 (1.25-2.23) | 122 (15.2%) | 2.60 (1.89-3.57) | 1.60 (1.37-1.86) |
| Well-being | 408 (8.1%) | 56 (5.3%) | 1 (ref) | 240 (7.8%) | 1.38 (1.03-1.85) | 99 (12.4%) | 2.17 (1.56-3.02) | 1.49 (1.26-1.76) |

Based on the income, participants were categorized into low (highest quintile), intermediate (quintiles 2 to 4), and high (lowest quintile) groups to characterize socioeconomic deprivation. Model was adjusted for age, sex, ethnicity, marital status, comorbidities, pre-pandemic mental health conditions, and lifestyle factors. HR for trend indicates the change in HR by one category increment in socioeconomic deprivation towards most deprived. HR=hazard ratio.

**Table S5. Association between deprivation category characterized by wealth and mental health conditions**

|  | **Total (n=5049)** | **Least deprived**  **(n=1232)** | | **Intermediate**  **(n=3103)** | | **Most deprived**  **(n=622)** | | **HR for trend (95% CI)** |
| --- | --- | --- | --- | --- | --- | --- | --- | --- |
|  |  | Events | HR | Events | HR (95% CI) | Events | HR (95% CI) |  |
| Depression | 1342 (26.6%) | 222 (18.0%) | 1 (ref) | 824 (26.6%) | 1.37 (1.21-1.54) | 270 (43.4%) | 2.17 (1.87-2.53) | 1.47 (1.36-1.59) |
| Anxiety | 466 (9.2%) | 56 (5.3%) | 1 (ref) | 276 (8.9%) | 1.40 (1.07-1.82) | 122 (15.2%) | 2.99 (2.22-4.04) | 1.79 (1.54-2.10) |
| Well-being | 408 (8.1%) | 56 (5.3%) | 1 (ref) | 240 (7.8%) | 1.42 (1.08-1.86) | 99 (12.4%) | 2.23 (1.60-3.09) | 1.50 (1.27-1.77) |

Based on the wealth, participants were categorized into low (highest quintile), intermediate (quintiles 2 to 4), and high (lowest quintile) groups to characterize socioeconomic deprivation. Model was adjusted for age, sex, ethnicity, marital status, comorbidities, pre-pandemic mental health conditions, and lifestyle factors. HR for trend indicates the change in HR by one category increment in socioeconomic deprivation towards most deprived. HR=hazard ratio.

**Table S6. Association between deprivation category characterized by education and mental health conditions**

|  | **Total (n=5049)** | **Least deprived**  **(n=1250)** | | **Intermediate**  **(n=2245)** | | **Most deprived**  **(n=1554)** | | **HR for trend (95% CI)** |
| --- | --- | --- | --- | --- | --- | --- | --- | --- |
|  |  | Events | HR | Events | HR (95% CI) | Events | HR (95% CI) |  |
| Depression | 1342 (26.6%) | 247 (19.8%) | 1 (ref) | 625 (27.8%) | 1.25 (1.11-1.41) | 470 (30.2%) | 1.45 (1.28-1.65) | 1.20 (1.13-1.28) |
| Anxiety | 466 (9.2%) | 76 (6.1%) | 1 (ref) | 218 (9.7%)） | 1.51 (1.17-1.97) | 172 (11.1%) | 1.86 (1.41-2.44) | 1.34 (1.18-1.52) |
| Well-being | 408 (8.1%) | 76 (6.1%) | 1 (ref) | 179 (8.0%) | 1.28 (0.98-1.67) | 153 (9.8%) | 1.50 (1.13-2.00) | 1.22 (1.06-1.40) |

Based on the education level, participants were categorized into low (high level), intermediate (middle level), and high (low level) groups to characterize socioeconomic deprivation. Model was adjusted for age, sex, ethnicity, marital status, comorbidities, pre-pandemic mental health conditions, and lifestyle factors. HR for trend indicates the change in HR by one category increment in socioeconomic deprivation towards most deprived. HR=hazard ratio.

**Table S7. Association between lifestyle category and mental health conditions by socioeconomic deprivation group characterized by income**

|  | **Total (n=5049)** | **Favorable lifestyle**  **(n=2101)** | | **Intermediate lifestyle**  **(n=2468)** | | **Unfavorable lifestyle**  **(n=480)** | | **HR for trend (95% CI)** |
| --- | --- | --- | --- | --- | --- | --- | --- | --- |
|  |  | Events | HR (95% CI) | Events | HR (95% CI) | Events | HR (95% CI) |  |
| Depression |  |  |  |  |  |  |  |  |
| Least deprived | 179/1060 (16.9%) | 81 (17.6%) | 1 (ref) | 84 (16.0%) | 1.08 (0.84-1.38) | 14 (19.2%) | 1.29 (0.82-2.02) | 1.11 (0.92-1.35) |
| Intermediate | 812/3096 (26.2%) | 302 (23.9%) | 1 (ref) | 405 (26.5%) | 1.17 (1.03-1.32) | 105 (34.2%) | 1.36 (1.22-1.66) | 1.17 (1.07-1.28) |
| Most deprived | 325/801 (40.6%) | 121 (36.6%) | 1 (ref) | 155 (41.0%) | 1.15 (0.93-1.42) | 49 (53.3%) | 1.59 (1.17-2.15) | 1.23 (1.06-1.43) |
| Anxiety |  |  |  |  |  |  |  |  |
| Least deprived | 56/1060 (5.3%) | 25 (5.4%) | 1 (ref) | 25 (4.8%) | 1.08 (0.61-1.90) | 6 (8.2%) | 1.78 (0.73-4.34) | 1.23 (0.81-1.87) |
| Intermediate | 276/3096 (8.9%) | 100 (7.9%) | 1 (ref) | 144 (9.4%) | 1.30 (1.00-1.68) | 32 (10.4%) | 1.44 (0.96-2.15) | 1.23 (1.02-1.47) |
| Most deprived | 122/801 (15.2%) | 41 (12.4%) | 1 (ref) | 61 (16.1%) | 1.28 (0.86-1.91) | 20 (21.7%) | 1.84 (1.07-3.18) | 1.34 (1.03-1.75) |
| Well-being |  |  |  |  |  |  |  |  |
| Least deprived | 56/1060 (5.3%) | 21 (4.6%) | 1 (ref) | 30 (5.7%) | 1.21 (0.68-2.13) | 5 (6.8%) | 1.40 (0.52-3.73) | 1.19 (0.78-1.83) |
| Intermediate | 240/3096 (7.8%) | 82 (6.5%) | 1 (ref) | 123 (8.1%) | 1.28 (0.97-1.70) | 35 (11.4%) | 1.84 (1.23-2.74) | 1.34 (1.10-1.63) |
| Most deprived | 99/801 (12.4%) | 31 (9.4%) | 1 (ref) | 45 (11.9%) | 1.08 (0.61-1.92) | 23 (25.0%) | 1.72 (0.85-3.46) | 1.64 (1.23-2.20) |

Based on the income, participants were categorized into low (highest quintile), intermediate (quintiles 2 to 4), and high (lowest quintile) groups to characterize socioeconomic deprivation. The favorable lifestyle category for each group of deprivation was used as the reference category. HR for trend indicates the change in HR by one lifestyle category change towards unfavorable. Model was adjusted for age, sex, ethnicity, marital status, comorbidities, and pre-pandemic mental health conditions and levels of loneliness and social isolation.

**Table S8. Association between lifestyle category and mental health conditions by socioeconomic deprivation group characterized by wealth**

|  | **Total (n=5049)** | **Favorable lifestyle**  **(n=2101)** | | **Intermediate lifestyle**  **(n=2468)** | | **Unfavorable lifestyle**  **(n=480)** | | **HR for trend (95% CI)** |
| --- | --- | --- | --- | --- | --- | --- | --- | --- |
|  |  | Events | HR (95% CI) | Events | HR (95% CI) | Events | HR (95% CI) |  |
| Depression |  |  |  |  |  |  |  |  |
| Least deprived | 222/1232 (18.0%) | 98 (18.0%) | 1 (ref) | 104 (17.2%) | 1.19 (0.95-1.49) | 20 (24.1%) | 1.05 (0.67-1.65) | 1.10 (0.93-1.31) |
| Intermediate | 824/3103 (26.6%) | 327 (24.9%) | 1 (ref) | 403 (26.8%) | 1.11 (0.98-1.26) | 94 (32.6%) | 1.31 (1.07-1.60) | 1.13 (1.04-1.24) |
| Most deprived | 270/622 (43.4%) | 79 (39.7%) | 1 (ref) | 137 (42.5%) | 1.08 (0.85-1.38) | 54 (53.5%) | 1.49 (1.09-2.04) | 1.20 (1.02-1.41) |
| Anxiety |  |  |  |  |  |  |  |  |
| Least deprived | 70/1232 (5.7%) | 32 (5.9%) | 1 (ref) | 32 (5.3%) | 0.93 (0.57-1.53) | 6 (7.2%) | 1.40(0.58-3.36) | 1.06 (0.72-1.57) |
| Intermediate | 262/3103 (8.4%) | 96 (7.3%) | 1 (ref) | 135 (9.0%) | 1.36 (1.04-1.77) | 31 (10.8%) | 1.57 (1.04-2.37) | 1.28 (1.07-1.54) |
| Most deprived | 122/622 (19.6%) | 38 (19.1%) | 1 (ref) | 63 (19.6%) | 1.13 (0.75-1.70) | 21 (20.8%) | 1.28 (0.74-2.21) | 1.13 (0.86-1.48) |
| Well-being |  |  |  |  |  |  |  |  |
| Least deprived | 67/1232 (5.4%) | 31 (5.7%) | 1 (ref) | 29 (4.8%) | 0.84 (0.50-1.40) | 7 (8.4%) | 1.50 (0.66 -3.42) | 1.05 (0.71-1.56) |
| Intermediate | 246/3103 (7.9%) | 81 (6.2%) | 1 (ref) | 132 (8.8%) | 1.44 (1.09-1.91) | 33 (11.5%) | 1.83 (1.22-2.76) | 1.37 (1.14-1.66) |
| Most deprived | 82/622 (13.2%) | 22 (11.1%) | 1 (ref) | 37 (11.5%) | 1.11 (0.65-1.91) | 23 (22.8%) | 2.34 (1.27-4.30) | 1.54 (1.11-2.13) |

Based on the wealth, participants were categorized into low (highest quintile), intermediate (quintiles 2 to 4), and high (lowest quintile) groups to characterize socioeconomic deprivation. The favorable lifestyle category for each group of deprivation was used as the reference category. HR for trend indicates the change in HR by one lifestyle category change towards unfavorable. Model was adjusted for age, sex, ethnicity, marital status, comorbidities, and pre-pandemic mental health conditions and levels of loneliness and social isolation.

**Table S9. Association between lifestyle category and mental health conditions by socioeconomic deprivation group characterized by education**

|  | **Total (n=5049)** | **Favorable lifestyle**  **(n=2101)** | | **Intermediate lifestyle**  **(n=2468)** | | **Unfavorable lifestyle**  **(n=480)** | | **HR for trend (95% CI)** |
| --- | --- | --- | --- | --- | --- | --- | --- | --- |
|  |  | Events | HR (95% CI) | Events | HR (95% CI) | Events | HR (95% CI) |  |
| Depression |  |  |  |  |  |  |  |  |
| Least deprived | 247/1250 (19.8%) | 107 (18.6%) | 1 (ref) | 117 (20.1%) | 1.13 (0.91-1.40) | 23 (24.7%) | 1.49 (1.03-2.13) | 1.18 (1.01-1.39) |
| Intermediate | 625/2245 (27.8%) | 260 (26.9%) | 1 (ref) | 303 (27.7%) | 1.08 (0.94-1.25) | 62 (33.5%) | 1.27 (1.00-1.63) | 1.11 (1.00-1.24) |
| Most deprived | 470/1554 (30.2%) | 147 (26.2%) | 1 (ref) | 237 (30.0%) | 1.24 (1.04-1.47) | 86 (42.6%) | 1.53 (1.21-1.94) | 1.24 (1.10-1.39) |
| Anxiety |  |  |  |  |  |  |  |  |
| Least deprived | 76/1250 (6.1%) | 33 (5.7%) | 1 (ref) | 36 (6.2%) | 1.24 (0.77-2.00) | 7 (7.5%) | 1.52 (0.67-3.46) | 1.24 (0.87-1.76) |
| Intermediate | 218/2245 (9.7%) | 86 (8.9%) | 1 (ref) | 109 (10.0%) | 1.23 (0.92-1.64) | 23 (12.4%) | 1.65 (1.03-2.64) | 1.26 (1.02-1.57) |
| Most deprived | 172/1554 (11.1%) | 52 (9.3%) | 1 (ref) | 91 (11.5%) | 1.31 (0.93-1.86) | 29 (14.4%) | 1.55 (0.98-2.45) | 1.25 (1.00-1.57) |
| Well-being |  |  |  |  |  |  |  |  |
| Least deprived | 76/1250 (6.1%) | 31 (5.4%) | 1 (ref) | 36 (6.2%) | 1.31 (0.91-1.87) | 9 (9.7%) | 1.32 (0.80-2.20) | 1.24 (0.87-1.78) |
| Intermediate | 179/2245 (8.0%) | 63 (6.5%) | 1 (ref) | 83 (7.6%) | 1.21 (0.87-1.70) | 33 (17.8%) | 3.14 (2.04-4.83) | 1.66 (1.32-2.09) |
| Most deprived | 153/1554 (9.8%) | 47 (8.4%) | 1 (ref) | 84 (10.6%) | 1.11 (0.65-1.91) | 22 (10.9%) | 2.34 (1.27-4.30) | 1.18 (0.93-1.50) |

Based on the education level, participants were categorized into low (high level), intermediate (middle level), and high (low level) groups to characterize socioeconomic deprivation. The favorable lifestyle category for each group of deprivation was used as the reference category. HR for trend indicates the change in HR by one lifestyle category change towards unfavorable. Model was adjusted for age, sex, ethnicity, marital status, comorbidities, and pre-pandemic mental health conditions and levels of loneliness and social isolation.


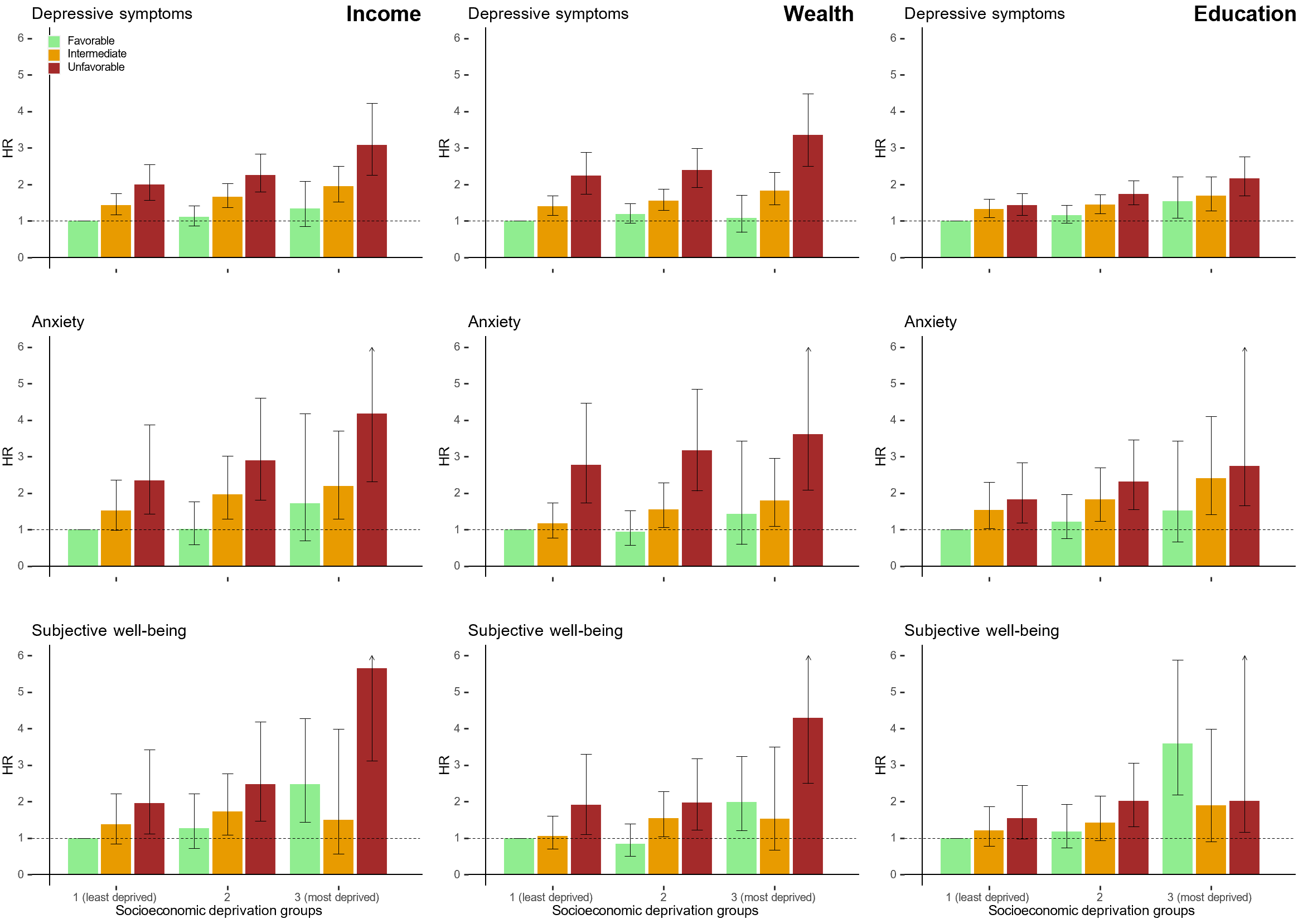


**Fig. S1.** Association between lifestyle category, socioeconomic deprivation group characterized by individual-level factors (income, wealth, and education), and mental health conditions. Model was adjusted for age, sex, ethnicity, marital status, comorbidities, and pre-pandemic mental health conditions. Error bars indicate 95% CIs. HR: hazard ratio. HR for trend indicates the change in HR by one lifestyle*deprivation category change towards unfavorable within each IMD category. Participants in the least deprived group who were in the favorable lifestyle category were defined as the reference group.


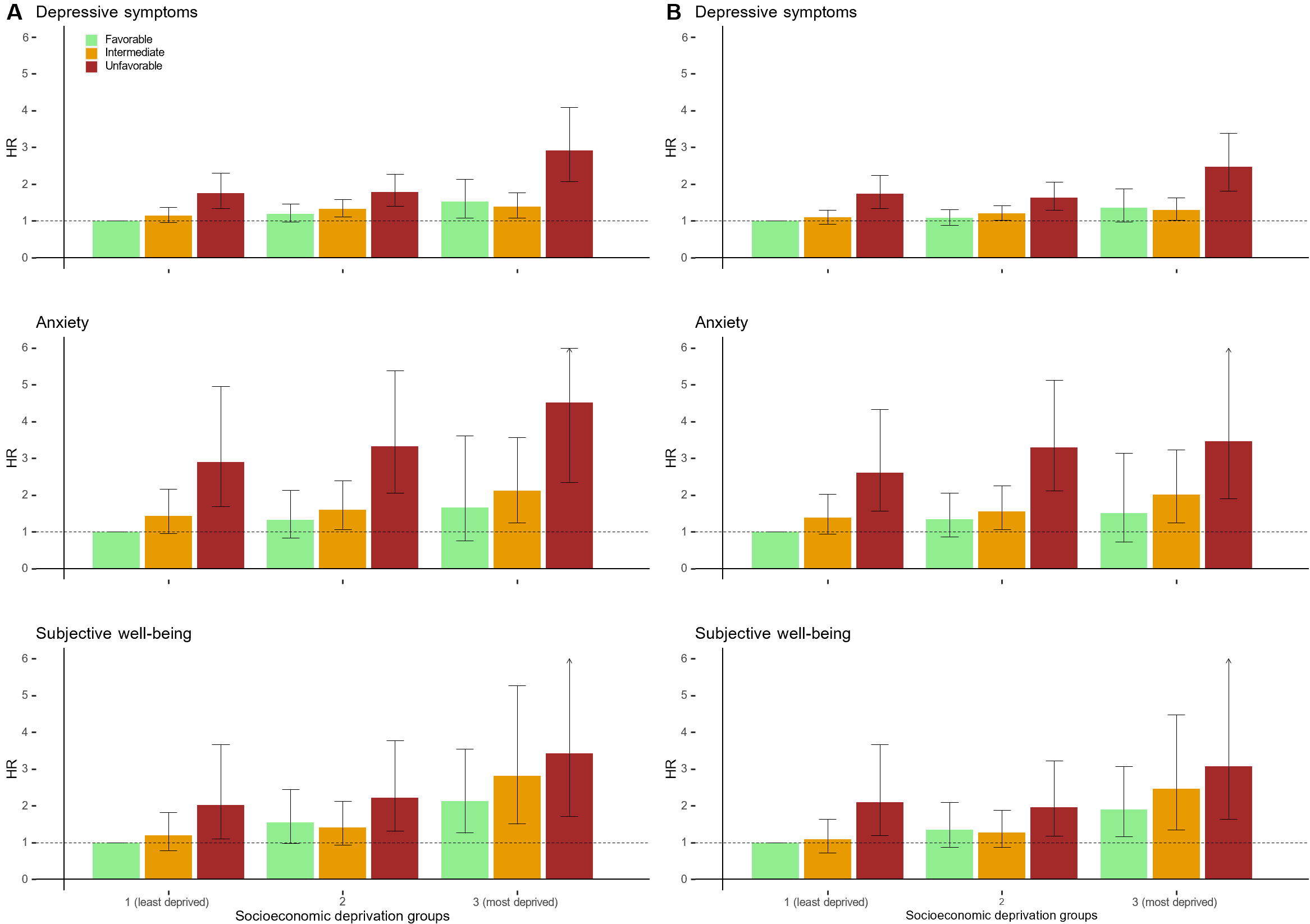

**Fig. S2.** Sensitivity analyses of the association between lifestyle category, socioeconomic deprivation, and mental health conditions. Model was adjusted for age, sex, ethnicity, marital status, comorbidities, and pre-pandemic mental health conditions. **(a)** Sensitivity analyses excluding those with history of diagnosed mental disorders. **(b)** Sensitivity analyses additionally adjusted for loneliness and social isolation. Error bars indicate 95% CIs. HR: hazard ratio. HR for trend indicates the change in HR by one lifestyle*deprivation category change towards unfavorable within each IMD category. Participants in the least deprived group who were in the favorable lifestyle category were defined as the reference group.
